# In silico evaluation of a new compound incorporating 4(3H)-quinazolinone and sulfonamide as a potential inhibitor of a human carbonic anhydrase

**DOI:** 10.1186/s13065-024-01150-1

**Published:** 2024-03-04

**Authors:** Ahmed M. Alkaoud, Abbas I. Alakhras, Moez A. Ibrahim, S. K. Alghamdi, Rageh K. Hussein

**Affiliations:** 1 Physics Department, College of Science, Imam Mohammad Ibn Saud Islamic University (IMSIU), 11623 Riyadh, Saudi Arabia; 2 Chemistry Department, College of Science, Imam Mohammad Ibn Saud Islamic University (IMSIU), 11623 Riyadh, Saudi Arabia; 3 Department of Physics, Taibah University, Al-Madinah Al-Munawarah, Saudi Arabia

**Keywords:** 2, 4(3H)-quinazolinone, Sulfonamide, DFT, IR, UV–vis spectra, ADMET, Molecular docking, Molecular dynamics

## Abstract

The present study investigates the potential of a new compound containing sulfonamide and 4(3H)-quinazolinone to inhibit the hCA-IIX enzyme using in silico methods. Density functional theory-based calculations of electronic properties have been addressed through the analysis of frontier molecular orbitals, molecule electrostatic potential, and IR and UV–vis spectroscopy data. A molecular electrostatic potential analysis predicts that the target protein will be most inhibited by the sulfonamide groups since it has the highest potential spots for electrophile and nucleophile attack. The investigated compound exhibited good ADMET properties and satisfied the Lipinski rule of drug likeness. The hCA-IIX protein binding affinity with the proposed compound was determined by molecular docking analysis, which revealed a stable conformation with more negative binding energy (−12.19 kcal/mol) than the standard AZA drug (−7.36 kcal/mol). Moreover, a molecular dynamics study confirmed the docking results through trajectory analysis. The RMSD and RMSF both showed convergence and no significant fluctuations during the simulation time, which revealed a stable interaction within the active domain of the target protein. According to these findings, the proposed compound has a good pharmacological nature and could potentially be an efficient drug against hCAIX enzymes.

## Introduction

Human carbonic anhydrase (hCA) isoforms range from hCA-I to hCA-XV, representing sixteen different isoforms. These isoforms are related to many essential biological processes and are widely distributed throughout various organs and tissue types [1–3]. hCA are divided into several groups based on their cellular localization, molecular characteristics, distribution within the human body, expression levels, and reactivity to different inhibitory classes [4, 5]. The impact of the reaction products produced by hCA can be regulated by using enzyme inhibitors. For example, to treat excessive water retention in the eyes (glaucoma), hCA inhibitors are used to control the bicarbonate ions that affect how much water is in the eyes [6]. As a result, inhibitors of several isoforms of hCA have been used in medical applications to treat a range of diseases, including cancer, epilepsy, retinopathy, hemolytic anaemia, and osteoporosis [7, 8]. Finding selective inhibitors for the isoform of hCA can be challenging because of its varied clinical effects. Currently, many hCA isoforms represent important targets for the development of selective inhibitors with potential clinical applications. The inhibitory action of recently synthesized molecules against hCA has been the subject of many recent studies. Newly synthesized substituted hydroxyl Schiff derivatives based on the quinazoline scaffold have been utilized to investigate the inhibitory effects of hCA [9]. Four isoforms of hCAI, hCAIX, and hCAI were the targets of screening for the inhibitory effects of two new series of sulphonamides based on isatin N-phenylacetamide [10].

The 4(3H)-quinazolinone ring structures are highly important heterocyclic compounds due to their potential for various biological activities [11]. Many 4(3H)-quinazolinone derivatives have been developed and investigated as prospective analgesic, anti-inflammatory, and anticancer drugs, as well as potent antibacterial agents [12–14]. The most significant feature is that the 4(3H)-quinazolinone derivatives have been shown to have considerable inhibition of cyclooxygenase 2 (COX-2) and thus anti-inflammatory behavior. The inclusion of sulfonamide substituents in one of para position of the phenyl rings in quinazolinone derivatives revealed specific COX-2 inhibitory efficacy [15, 16]. Strong COX-2 inhibition activities by 2,3-disubstituted-4(3H)-quinazolinone with a p-benzene sulfonamide moiety positioned at the phenyl ring were reported by a previous molecular docking study [17]. In addition to the above-indicated properties, a variety of quinazolinone compounds stand out as potent hCA inhibitors. At the nano-molar level, several derivatives of substituted-4 (3H)-quinazolinones proved considerable inhibition capability for α-CA, as well as hCA-I and hCA-II [18].

In the trend of drug design and discovery, in silico investigations of biological activities have been shown to be capable of effectively identifying active compounds. Structure-based methodologies, including ADMET profiles, molecular docking, and molecular dynamics, have produced reliable molecular data relevant to the therapeutic behaviors of tested molecules [19–21]. Prior studies confirmed the strong correlation between biological activity and the corresponding electronic properties calculated by density functional theory (DFT) [22]. In search of competitive inhibitors of hCA-II, Yazg Dizdaroglu and others designed and evaluated a number of pyrazole compounds as carbonic anhydrase inhibitors using a molecular modelling approach [23]. Another investigation on the inhibition effect of carbonic anhydrase was carried out by Mackenzie Taylor and Junming, who employed computational methodologies to predict relative binding affinities for a set of structurally differing ligands [24].

The current investigation aims to study new sulfonamide-substituted diarylheterocycles compound (as a 4(3H)-quinazolinone derivative) using molecular modelling simulations to explore its inhibitory capabilities against hCA. Both drug-likeness and ADMET features were used to determine the probability of having oral bioavailability and the appropriate pharmacokinetic parameters. The inhibitory action of human carbonic anhydrase (hCA-IIX) by the stated compound was tested by molecular docking analysis. Additionally, the protein–ligand complex was subjected to molecular dynamics simulation to give an insight into the dynamic performance at the active site of the target protein. This study could be used as a foundation for future investigations into such types of compounds; 4(3H)-quinazolinone includes sulfonamide substituents. A number of characteristics and mechanisms identified by the results provided indicate that efforts to develop effective hCA inhibitors might require a new start with the incorporation of sulfonamide groups into quinazolinone derivatives.

## Materials and methods

### Chemistry

**Fig. 1:**
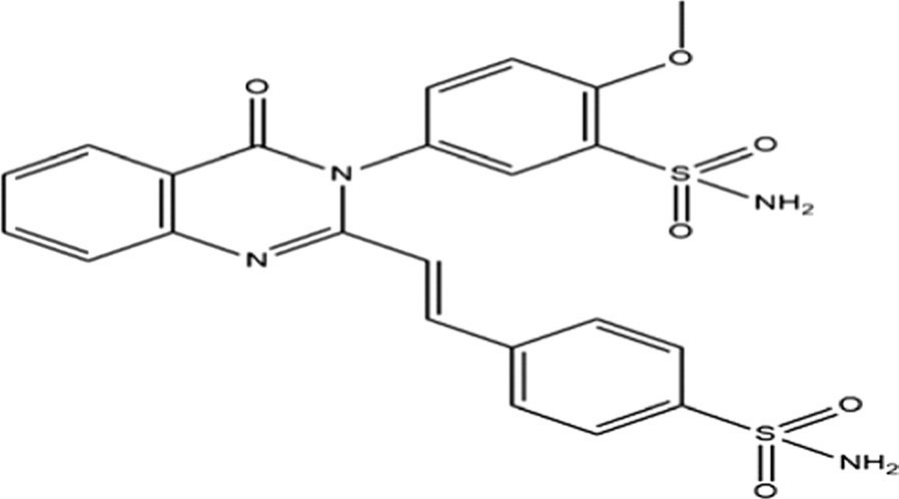
Molecular structure of 2-methoxy-5-{4-oxo-2-[(E)-2-(4-sulfamoylphenyl) ethenyl-3, 4-dihydroquinazolin-3-yl] benzene-1-sulfonamide compound, that will abbreviate as SSDC

The A new compound named 2-methoxy-5-{4-oxo-2-[(E)-2-(4-sulfamoylphenyl) ethenyl-3, 4-dihydroquinazolin-3-yl] benzene-1-sulfonamide was obtained from an earlier study that was reported in the Molbank database [25]. The objective was to create many-membered rings as heterocycle compounds, such as 4(3H)-quinazolinone and sulfonamide rings, which are widely regarded as promising COX-2 inhibitors. The title compound, which has the molecular structure shown in Fig. 1, was synthesized by utilizing chlorosulfonation-amidation on the starting material 3-(4-methoxyphenyl)-2-styryl-4(3H)-quinazolinone. The IR, 1H-NMR, 13C-NMR, and mass spectral data were used in that study to confirm the structure of the synthesized compound. SSDC will be used as the acronym for the compound under study since it is a sulfonamide-substituted diarylheterocycle compound.

### Computational methodology

The optimized structure of SSDC was obtained by using DFT calculations with the B3LYP/6-311G (d, p) level of theory executed using Gaussian 09 software [26]. The Avogadro visualization tool was used to visualize the frontier molecular orbitals and molecular electrostatic potential [27]. The same model was employed to calculate vibrational frequencies, while the UV–vis spectrum in the gas phase was obtained using the TDDFT method. The physicochemical characteristics that were associated with drug likeness parameters for the SSDC molecule were evaluated using the online webserver Molinspiration (https://www.molinspiration.com/). The standard pharmacokinetics were estimated using the integrated online tool for predicting ADMET properties (http://qsar.chem.msu.ru/admet/).

The Auto Dock 4.2.6 software was used to carry out the molecular docking study [28]. The target protein, with the PDB code 5FL4, was employed, and the 3D crystal structure was obtained via the Protein Data Bank (https://www.rcsb.org/). The protein structure was cleaned by removing any bonded ligand groups and water molecules, and then polar hydrogen atoms were added. The energy-minimized structure of the SSDC ligand and the prepared protein were saved in PDBQT format. The dimensions of the grid box were defined from the PDB site records of the target protein. Molecular docking was performed using the Lamarckian Genetic Algorithm with a total of 100 docking runs and default settings for other docking parameters. LigPlot + v1.4.5 and Discovery Studio version 2017 were used to analyze and visualize the docking results [29, 30].

On the basis of the docking information, further molecular dynamics were applied to the complex pose that had the lowest energy. NAMD 2.13 software (Nanoscale Molecular Dynamics) was used to simulate molecular dynamics [31]. The parameters of the ligand with the CHARMM force field were obtained from the input generator of the CHARMM-GUI (https://www.charmm-gui.org/), and visual molecular dynamics (VMD) software [32] was used to generate all protein parameter files. The protein–ligand complex was placed in the center of a TIP3P water molecule cubic box, and to maintain the system's neutrality, Na + and Cl-ions in the amount of 0.15 M were added. After being minimized, annealed, and equilibrated, the system was subjected to 100-ns molecular dynamics simulations using a Martyna-Tobias-Klein barostat set to 1 bar of pressure and a Nose–Hoover thermostat set to 300 K under NPT conditions.

## Results and discussion

### HOMO&LUMO analysis

**Fig. 2:**
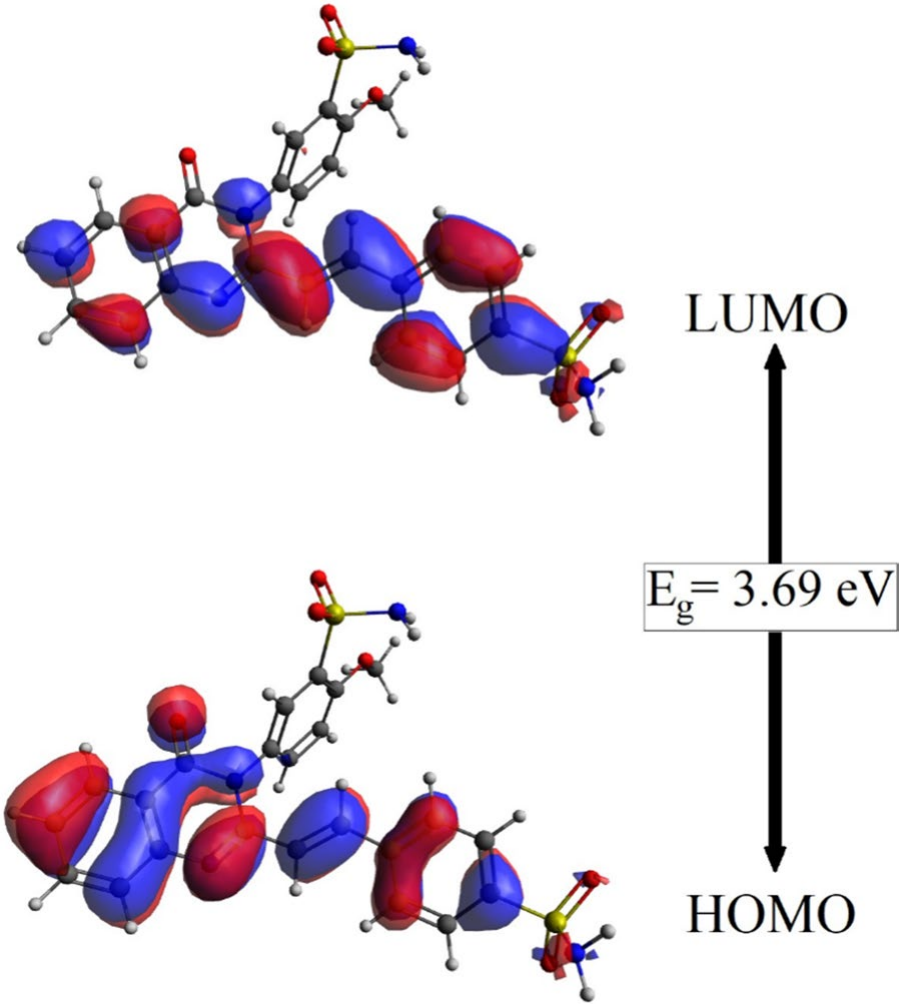
The HOMO and LUMO distribution pattern of SSDC

Chemical reactivity is known to be directly affected by frontier orbitals, often known as frontier molecular orbitals (FMO). The ability to provide an electron is denoted by the HOMO, while the ability to receive an electron is described by the LUMO. Intramolecular charge transfer (ICT) is primarily affected by two orbitals: the highest occupied molecular orbital (HOMO) and the lowest unoccupied molecular orbital (LUMO). The energy gap between the HOMO and LUMO levels was found to be 3.69 eV. The distribution of HOMO and LUMO frontier molecular orbitals throughout the surface of SSDC is shown in Fig. 2. The HOMO is extended bonding orbitals on the linear chain of benzene, ethenyl, and dihydroquinazolin. LUMO are localized on the same chain, with a bonding character between adjacent units and. an antibonding character within each unit. Methoxy, sulfamoyl-phenyl, and sulfonamide have no contribution to either HOMO or LUMO*.*

### Molecular electrostatic potential analysis

**Fig. 3:**
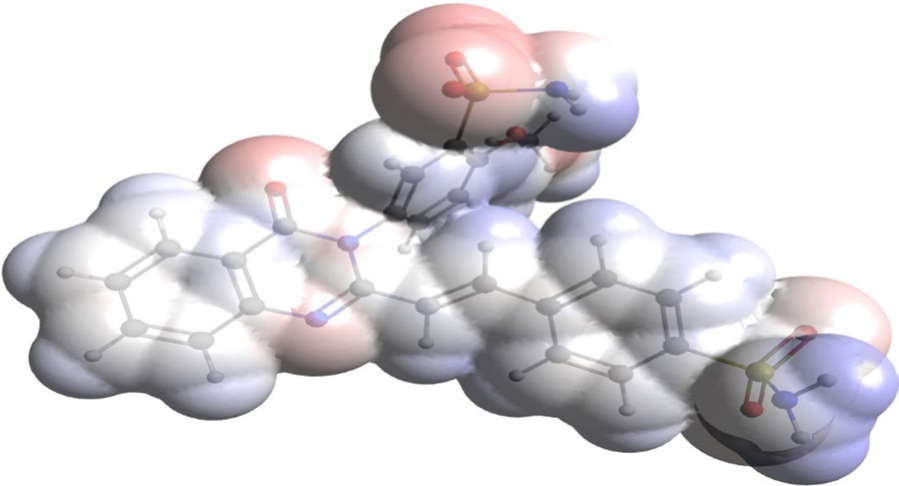
Three-dimensional molecular electrostatic potential surface map for SSDC

The molecular electrostatic potential (MEP) is commonly used to identify a molecule's reactivity sites for interactions with other compounds. MEP descriptor could be very helpful in identifying locations in chemical compounds for nucleophilic and electrophilic reactions in order to explore their biological activity mechanisms when interacting with active sites of the macrostructures. Surface study of MEP provides valuable insights into the biological processes of molecular structures by visualizing their charged regions as a map of electron excess and deficiency. Blue and red color portions in the MEP mapping correspond to the regions with positive and negative electrostatic potential, whereas white color parts represent the regions with zero potential. Nucleophilic and electrophilic attacks are most likely to occur at sites of positive and negative electrostatic potential, respectively. The MEP-mapped surface of the studied compound is depicted in Fig. 3.

The hydrogens of the sulfonamide groups constituted the most positive MEP regions, were distinguished by a denser blue color, and therefore are susceptible to nucleophilic attacks. On the other hand, the oxygen in sulfonamide groups showed the denser red color, which indicates that they constitute the most negative sites and thus are more susceptible to electrophilic attack. Moreover, a small negative potential with a less intense red color is shown by the oxygen and nitrogen atoms in the dihydroquinazolin group. Based on the obtained MEP results, the constituent atoms of the two sulfonamide groups in SSDC are the most active sites and are strongly nominated to participate in ligand-receptor interactions.

### IR spectra

**Fig. 4:**
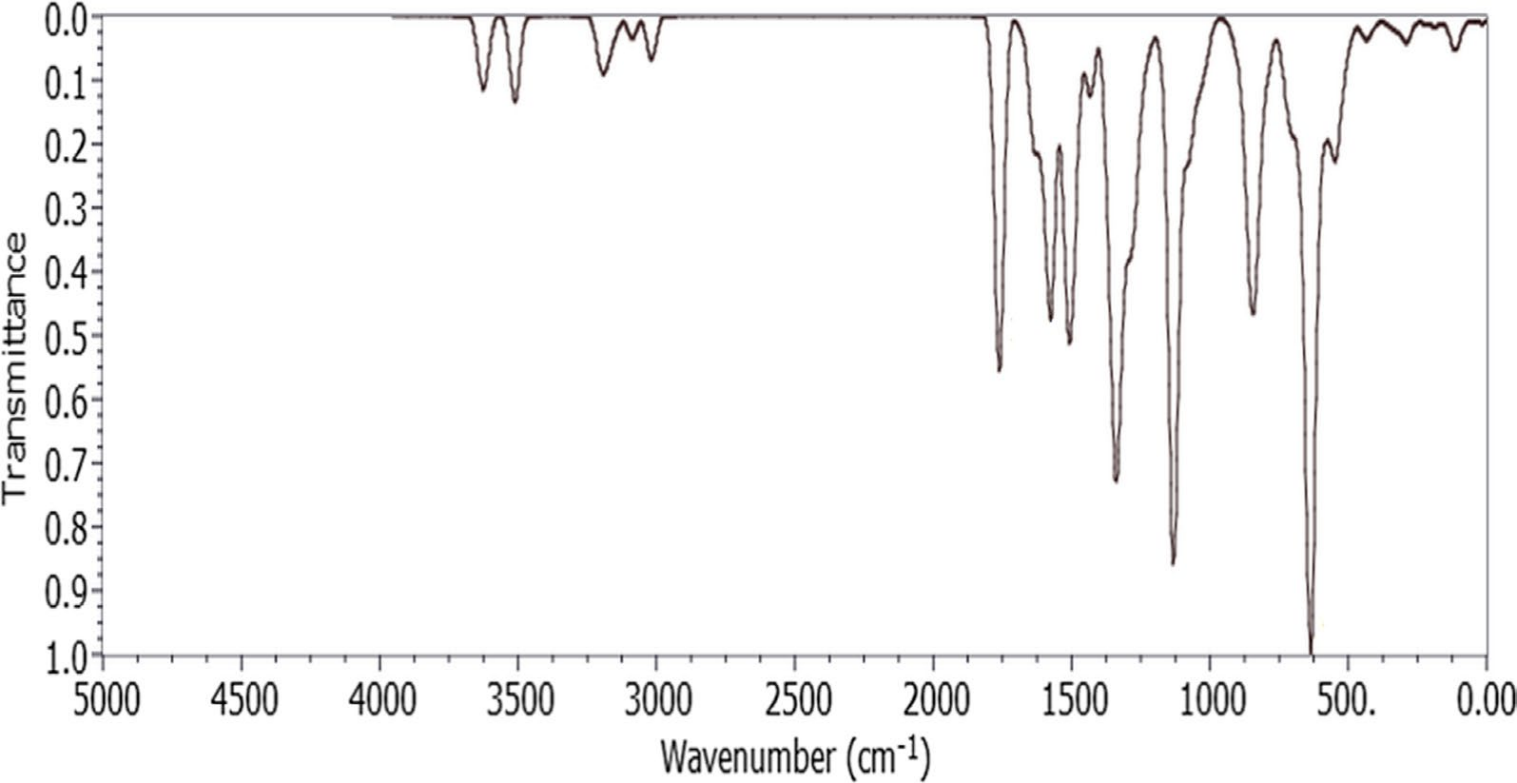
Calculated IR spectrum of SSDC at DFT (B3LYP)/6-311G (d, p) level

The vibrational frequencies and their assignment modes have been calculated using the same computational model, B3LYP/6-311G (d, p). The calculated frequencies were scaled to more closely match repeatable experimental data using the appropriate scaling factor due to the known overestimation in the DFT calculation of the vibrational normal modes [33]. Figure 4 displays the theoretical IR spectra of the studied compound. Table 1 summarizes the values of the selected bands, their vibration assignment, and the available experimental values.

**Table 1:** A selection of the calculated vibrational frequencies along with their assigned modes and the available corresponding experimental values

No;	Calculated frequencies (cm^−1^)		Experimental frequencies (cm^−1^) [24]	Assignment
	Unscaled	Scaled		
1	630.98	600.69	–	ω (N–H)
2	641.93	611.12	–	ω (N–H)
3	842.38	801.95	–	ω (C–H) R
4	1133.89	1079.47	–	α(C–H) R
5	1208.59	1150.58	–	α(C–H) R
6	1231.34	1172.24	1161	ν (S = O) s
7	1328.37	1264.61	1270	γ (C–H)
8	1433.17	1364.37	1338	ν (S = O) as
9	1645.51	1566.52	1607	ν (C = N)
10	1684.15	1603.31	1550	ν (C = C)
11	1764.57	1679.87	1663	ν (C = O)
12	3021.04	2876.03	–	ν (C–H) s
13	3090.57	2942.22	–	ν (C–H) as
14	3149.78	2998.59	–	ν (C–H) as
15	3171.03	3018.83	–	ν (C–H) R as
16	3171.58	3019.35	–	ν (C–H) R as
17	3181.79	3029.06	–	ν (C–H)
18	3186.32	3033.38	–	ν (C–H) R as
19	3190.69	3037.53	–	ν (C–H) R as
20	3191.45	3038.26	–	ν (C–H) R as + ν (C–H)
21	3199.27	3045.71	–	ν (C–H) R as
22	3203.26	3049.5	2945	ν (C-H)
23	3205.51	3051.65	–	ν (C–H) R as
24	3206.41	3052.50	–	ν (C–H) R s
25	3212.75	3058.54	–	ν (C–H) R as
26	3255.94	3099.66	3069	ν (C-H) R s
27	3513.29	3344.65	–	ν (N–H) s
28	3520.45	3351.47	3235	ν (N–H) s
29	3621.72	3447.88	3381	ν (N–H) as
30	3637.7	3463.09	–	ν (N–H) as

*R* Ring, *s* symmetric, *a* asymmetric, *ν* stretching, *γ* rocking, *α* scissoring, *twist* twisting, *ω* wagging

The literature reports that the N–H stretching vibration can be found in the IR spectra at 3300–3500 cm^−1^ [34]. Two symmetric and two asymmetric (N–H) stretching vibrations were detected at 3463.09, 3447.88, 3351.47, and 3344.65 cm^−1^, respectively. The observed experimental values, which were symmetric (3235 cm^−1^) and asymmetric (3381 cm^−1^), were extremely close to the calculated values. The aromatic ring of the sulfamoyl-phenyl had a C-H stretching that was determined at 3099.66 cm^−1^, whereas the experimental value was found at 3069 cm^−1^. A further asymmetric C-H stretching of the same aromatic ring was identified at 3058.54 cm^−1^. The other aromatic ring of the second sulfamoyl-phenyl has been found to exhibit both symmetric and asymmetric C-H stretching vibration at 3051.65and 3212.75 cm-1, respectively. The observed alkyl C-H stretching value of 2945 cm^−1^ was in conformity with the theoretical value of 3049.5 cm^−1^. The methoxy group was found to have a noticeable C-H stretching band at 2876.03 cm^−1^. The stretching mode of C = O is predicted to occur between 1850 and 1550 cm-1, according to previous studies [35]. The calculated stretching mode of vibration of C = O has been determined at 1679.87 cm^−1^, while the experimentally observed value was 1663 cm^−1^. The stretching vibrational mode of C = C group was found to have theoretical and experimental values of 1603.31 and 1550 cm-1, respectively. Theoretical data indicates the C = N stretching vibration at 1566.52 cm^−1^, and experimental data shows it at 1607 cm^−1^. SO2 asymmetric and symmetric stretching vibrations were detected at 1364.37 and 1172.24 cm ^−1^ with corresponding experimental values at 1338 and 1161 cm^−1^. Ar–O-C group's wagging mode was determined at 1328.37 cm^−1^ with an experimental value recorded at 1264.61 cm^−1^. The highest peak of the IR spectra in Fig. 4 is attributed to the strong NH2 wagging mode, which is located at 611.12 cm^−1^. Based on the above analysis, a good agreement was found between the scaled wavenumbers and the experimental spectra.

### UV − Vis study

D-DFT calculations were used for simulating the excited-stated structures and the absorption spectra of SSCD in the gas phase. The maximum absorption wavelength (λ _max_), oscillator strength (f), and the major contributions of the calculated electronic transition are displayed in Table 2.

**Table 2:** The electronic transition parameters of SSCD

No	λ _max_ (nm)	Osc. Strength	Major	Contributions (%)
1	364.081	1.000	HOMO → LUMO	97
2	335.355	0.1047	H-1 → LUMO	94

**Fig. 5:**
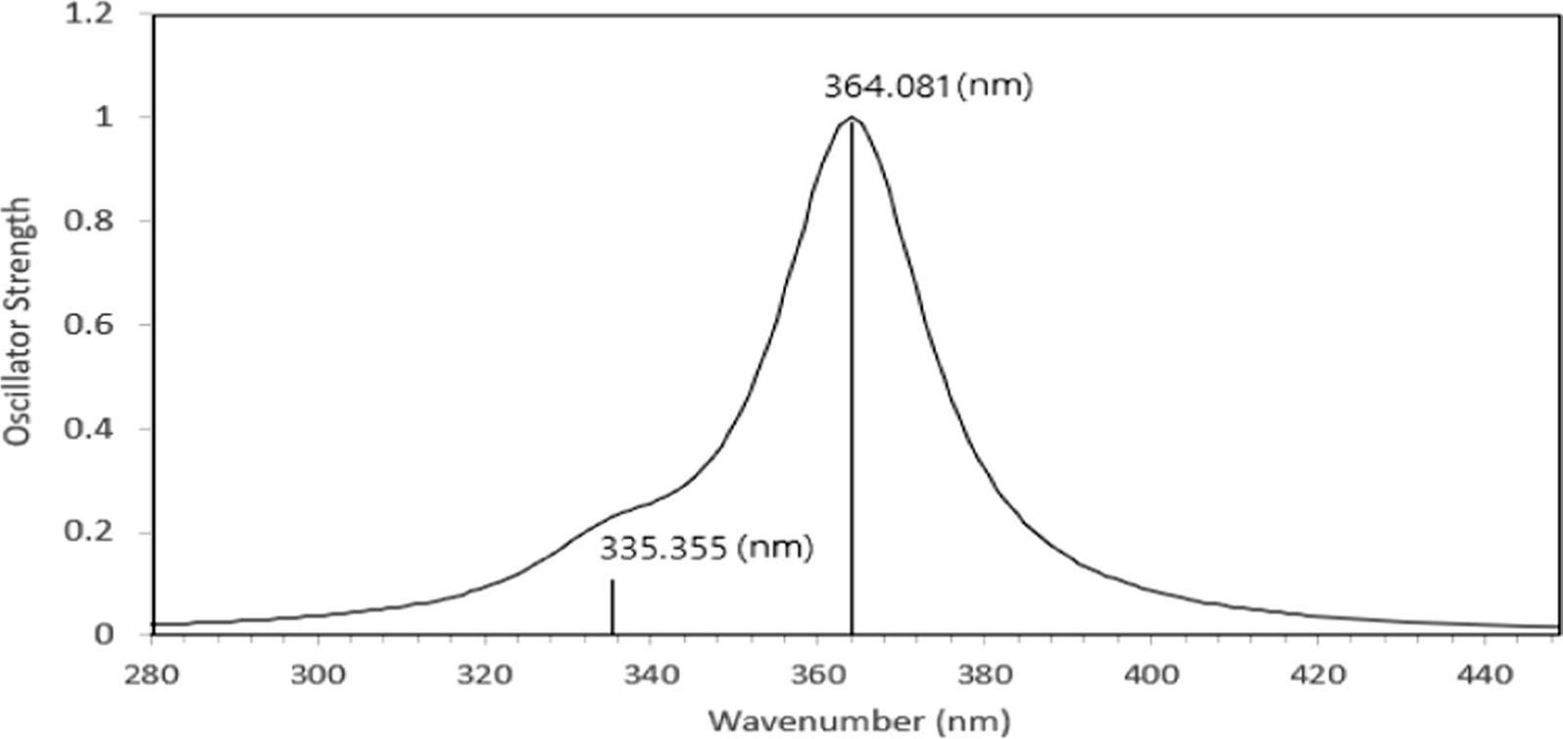
The absorption spectra of SSCD calculated using TD-DFT with the B3LYP /6-311G model

Figure 5 displays the absorption spectra of SSCD, which indicate the presence of two absorption bands at corresponding wavelengths of 335.355 and 364.081 nm. The strong peak at 364.081 nm is attributed to the HOMO → LUMO transition, with a major contribution of 97%. This electronic transition can be assigned to an intramolecular charge transfer (ICT). The π–π* electron transition is represented by the weak band at 335.355 nm. The higher oscillator strength value at the 364.081 nm band indicates that a larger number of electrons are involved in the transition from ground to excited state.

### Physicochemical parameters and ADMET properties

Both the efficacy and the safety of a drug are determined by its physicochemical properties, which are related to the drug's oral bioavailability. Lipinski's "Rule of Five" was used as a framework for evaluating if SSDC complied with the parameters for a drug-likeness character. Lipinski's rule clarifies the criteria limit for molecular weight, the octanol/water coefficient (log P), and hydrogen bond donors/acceptors (HBD/HBA), with the condition of no more than one violation [36]. In Table 3, there is just one exception to Lipinski's rule for SSDC: the molecular weight exceeded the acceptable limit < 500. In addition, the number of rotatable bonds (nrob) and water solubility were included in Table 3, and their computed values confirmed the oral bioavailability of SSDC.

**Table 3:** Physicochemical characteristics of SSDC and the upper limit for Lipinski's "Rule of Five"

MW (g/mol)	cLogP	HBD	HBA	Water solubility	nrob
< 500	≤ 5	≤ 5	≤ 10	–	≤ 10
512	1.77	4	10	−4.87	6

**Fig. 6:**
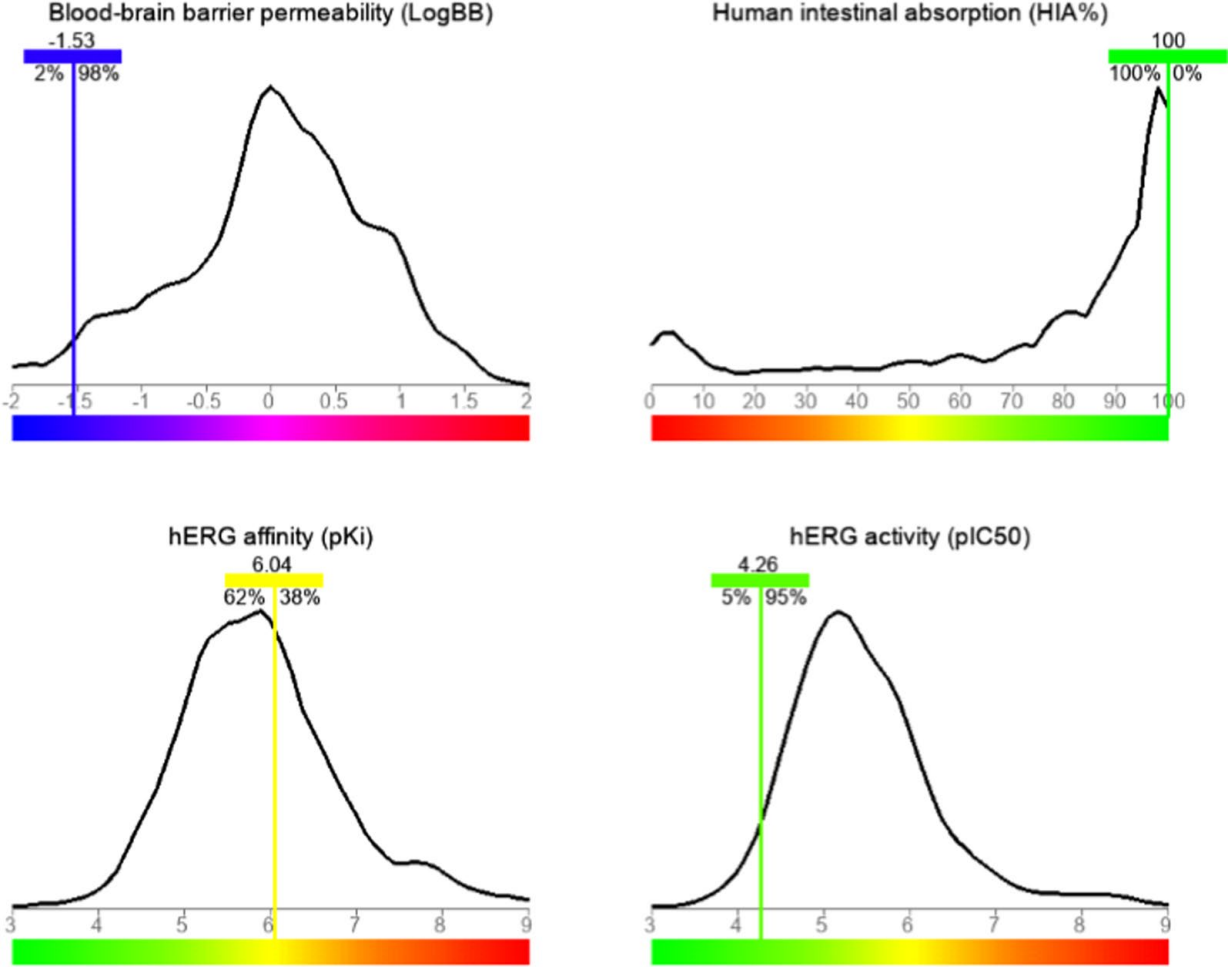
Four components that express the ADMET profile for the SSDC

Compounds A system for predicting the pharmacokinetic characteristics and toxicity of drug compounds is provided by the integrated online service for ADMET property prediction. The reduced list of ADMET features for this system includes four parameters: blood-brain barrier (BBB) permeability (log BB), human intestinal absorbance (HIA), hERG activity (pIC50), and hERG affinity (pKi). The system utilizes a previously calculated curve for the ADMET properties of a broad collection of organic compounds. The peak of this curve denotes the greatest number of the tested organic molecules that met the corresponding pharmacokinetic value. Also, the graph includes color-coded distribution, the favorite pharmacokinetic properties when leaning more toward blue or green, and unfavorable ones when leaning closer toward red. As shown in Fig. 6, a good oral absorption for SSDC was detected by the highest human intestinal absorbance (HIA) value that exceeded all other compounds on the curve. The blood-brain barrier (BBB) is crossed by molecules with log BB > 0.3, whereas those with log BB < −1 have poor distribution in the brain [37]. A poor penetration of BBB for SSDC was indicated by a log BB score of −1.53. During the development of new drugs, reducing the activity against the hERG channel is an essential aspect. Drugs are likely to exhibit some disturbance associated with hERG channels if the pIC50 value is > 5 [38]. By obtaining a pIC50 value of 4.26, SSDC exhibited low inhibition against hERG channels. A drug's affinity to bind the hERG channel is measured by its pKi value, which correlates to the dissociation constant Ki (pki = log 1/Ki). With a pKi value of 6.04, which is within the safe pharmacological range (5≤ pKi ≤ 8), SSDC is classified as an inactive compound with respect to the hERG channel.

### Molecular docking

The co-crystallized ligand 5-(1-naphthalen-1-yl-1,2,3-triazol-4-yl) thiophene-2-sulfonamide is always found in complex with the 3D structure of the 5FL4 protein that was downloaded from the Data Bank website. Therefore, the co-crystallized ligand was redocked into the active site of the 5FL4 protein in order to evaluate the effectiveness of the docking procedure. This is referred to as molecular docking validation, in which the ligand was properly combined with the active site with an −7.54 kcal/mol binding energy and an RMSD of 1.305 Å.

The docking results were assessed with regard to the binding energy, inhibition constant, and number of hydrogen bonds. These parameters are individually listed in Table 4. SSDC obtained a strong binding energy (− 12.19 kmol/cal) that is significantly higher than other prior findings, even than approved drugs. The binding energies for similar benzenesulfonamide compounds in earlier molecular docking studies ranged from  − 6.16 to − 8.15 kcal/mol [39]. Acetazolamide (AZA), the known Carbonic Anhydrase inhibitor, was docked into the same target protein as a standard reference. The binding energy recorded by AZA (− 7.36 kcal/mol) is small compared to SSDC. The studied compound exhibits stronger inhibition since its inhibition constant is lower than that of the AZA drug. The number of hydrogen bonds forming during the interaction between the ligand and the protein is a sign of high binding affinity. As shown in Table 4, SSDC formed seven conventional hydrogen bonds within the cavity of the 5FL4 protein, whereas five hydrogen bonds were produced in the interaction with the AZA drug.

**Table 4:** Docking results for SSDC and AZA with the target protein of human carbonic anhydrase 5FL4

Antimicrobial activity	Target protein	Binding energy (kcal/mol)	Inhibition constant (µM)	Hydrogen bonds	Interacting residues	Bond distance (Å)
SSDC	5FL4	-12.19	1.15 × 10–3	7	Val130(A)	3.00
					Arg129(A)	3.08
					Asp131(A)	2.84
					Asp131(C)	3.32
					Arg129(C)	2.69
					Arg129(C)	3.29
					Leu91(A)	2.83
AZA	5FL4	-7.36	4.02	5	Arg129(C)	2.90
					Asp131(C)	3.06
					Arg129(A)	3.22
					Val130(A)	3.01
					Ala128(C)	2.88

**Fig. 7:**
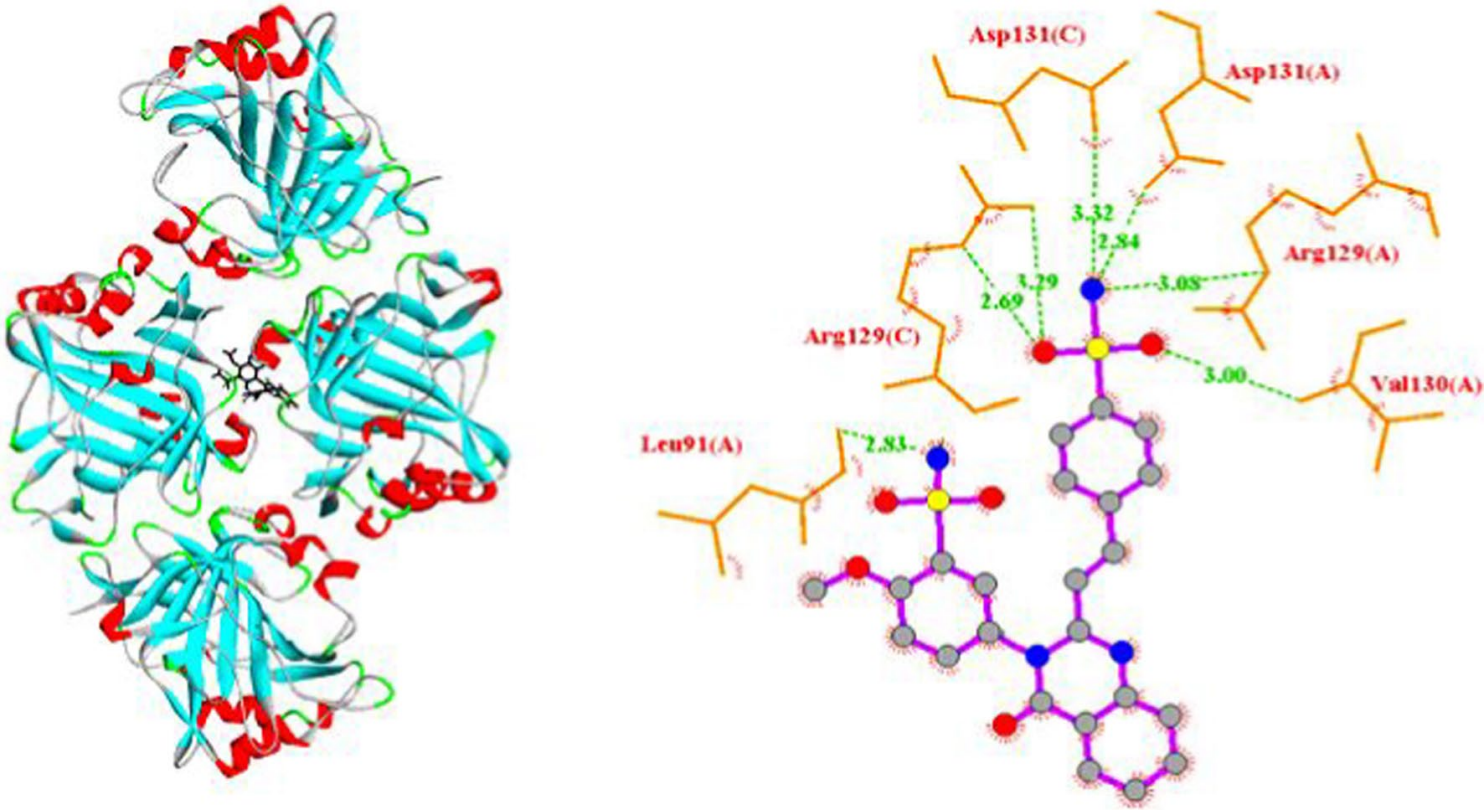
Three- and two-dimensional illustrations for SSDC in the protein cavity and hydrogen bond modes

**Fig. 8:**
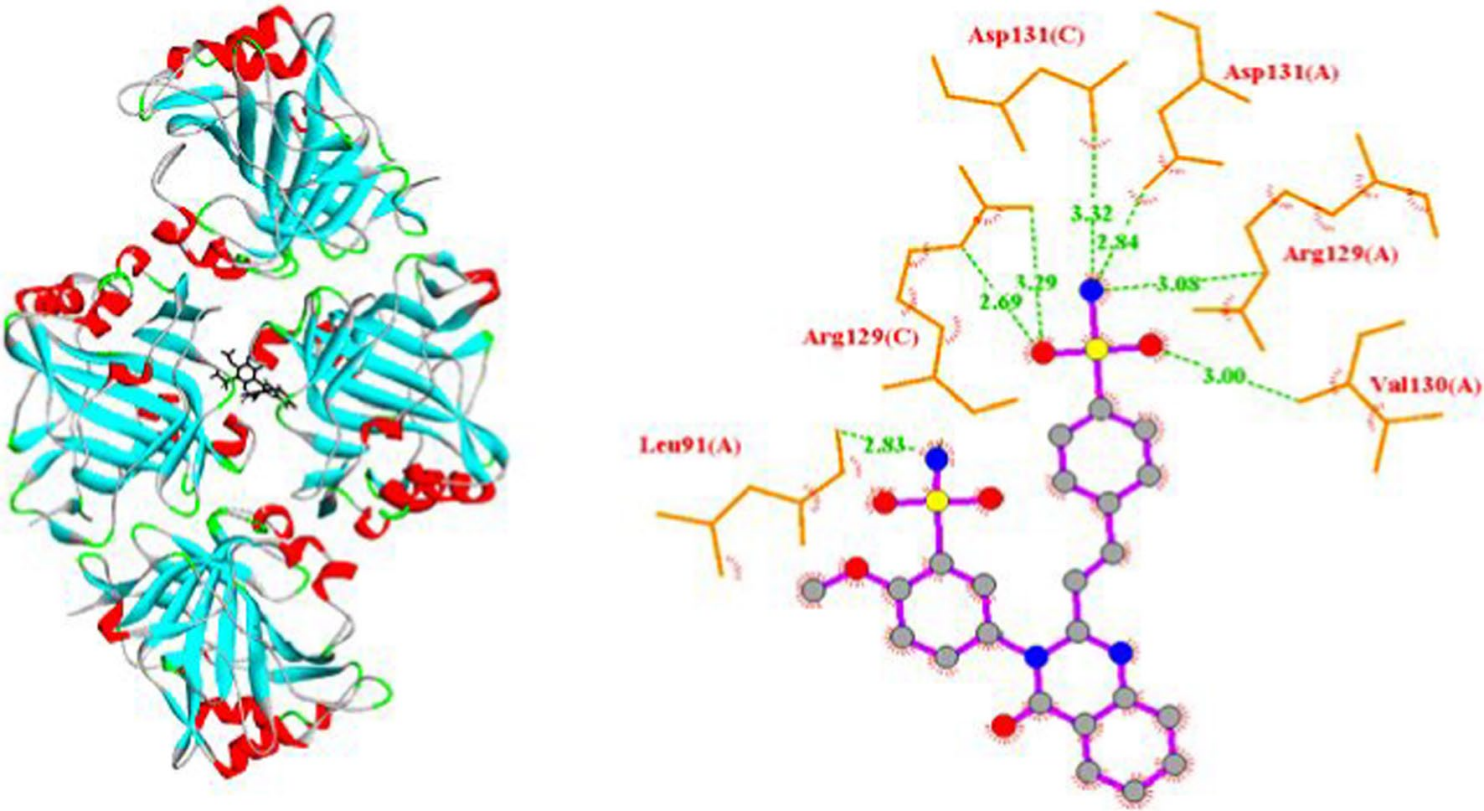
Three- and two-dimensional illustrations for AZA in the protein cavity and hydrogen bond modes

Figures 7 and 8 depict the molecular binding modes of SSDC and AZA at the binding site of the target protein. Both figures demonstrate the vital role of the sulfonamide group in the interaction with the target protein. In our proposed compound, the sulfonamide group was responsible for the formation of six of the seven hydrogen bonds. Four of the five hydrogen bonds formed in the case of AZA were provided by the sulfonamide group. This is viewed as a validation of the function played by sulfonamides as an important category of drugs in their capacity to inhibit carbonic anhydrases, as proven by many earlier studies [40].

### Molecular dynamics

**Fig. 9:**
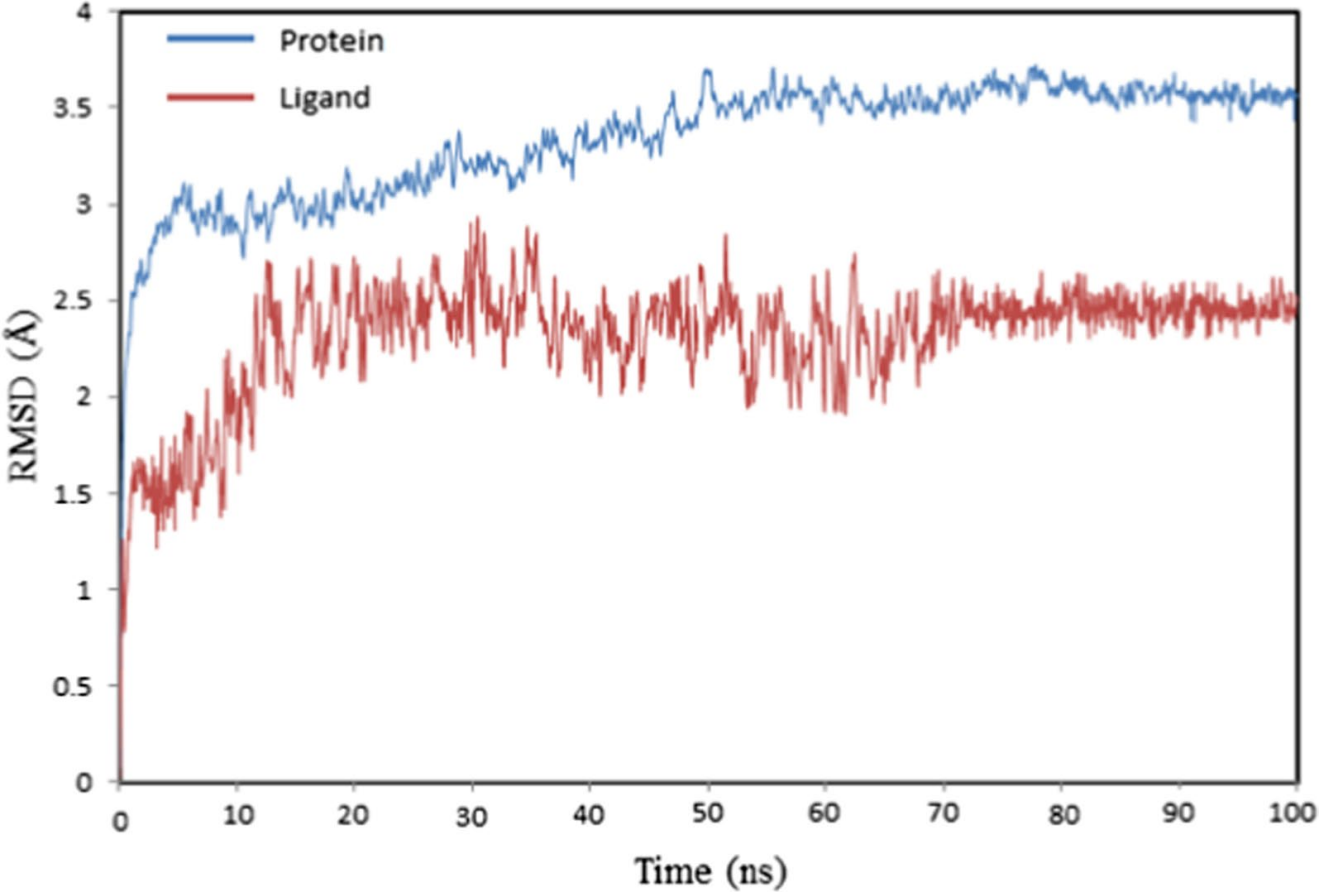
RMSD plot for the protein–ligand complex during 100 ns molecular dynamics simulations

The trajectory for the molecular dynamics simulation was analyzed using the root mean square deviation (RMSD) and root mean square fluctuation (RMSF) in order to evaluate the stability of the docked conformation between the targeted protein and ligand. The RMSD of the protein and ligand plot (Fig. 9) did not include any significant variation throughout the simulation period. For the protein, the first 3 ns showed a sharp increase from 0 to 2.5 Å. then the deviation value increased gradually from 2.5 to 3.5 Å until 60 ns. The complex steadily stabilized throughout the duration of the last 40 ns, attaining an equilibrium value of 3.5 Å. The RMSD of the ligand kept rising to 2.5 Å within the first 15 ns. Subsequently, the deviation showed an oscillation with an average value of 2.35 Å between 15 and 70 ns and achieved stable behavior at the final 30 ns at 2.5 Å. These outcomes show that the compound has a stable structure and that the ligand is maintained bound to the protein during the simulation time.

**Fig. 10:**
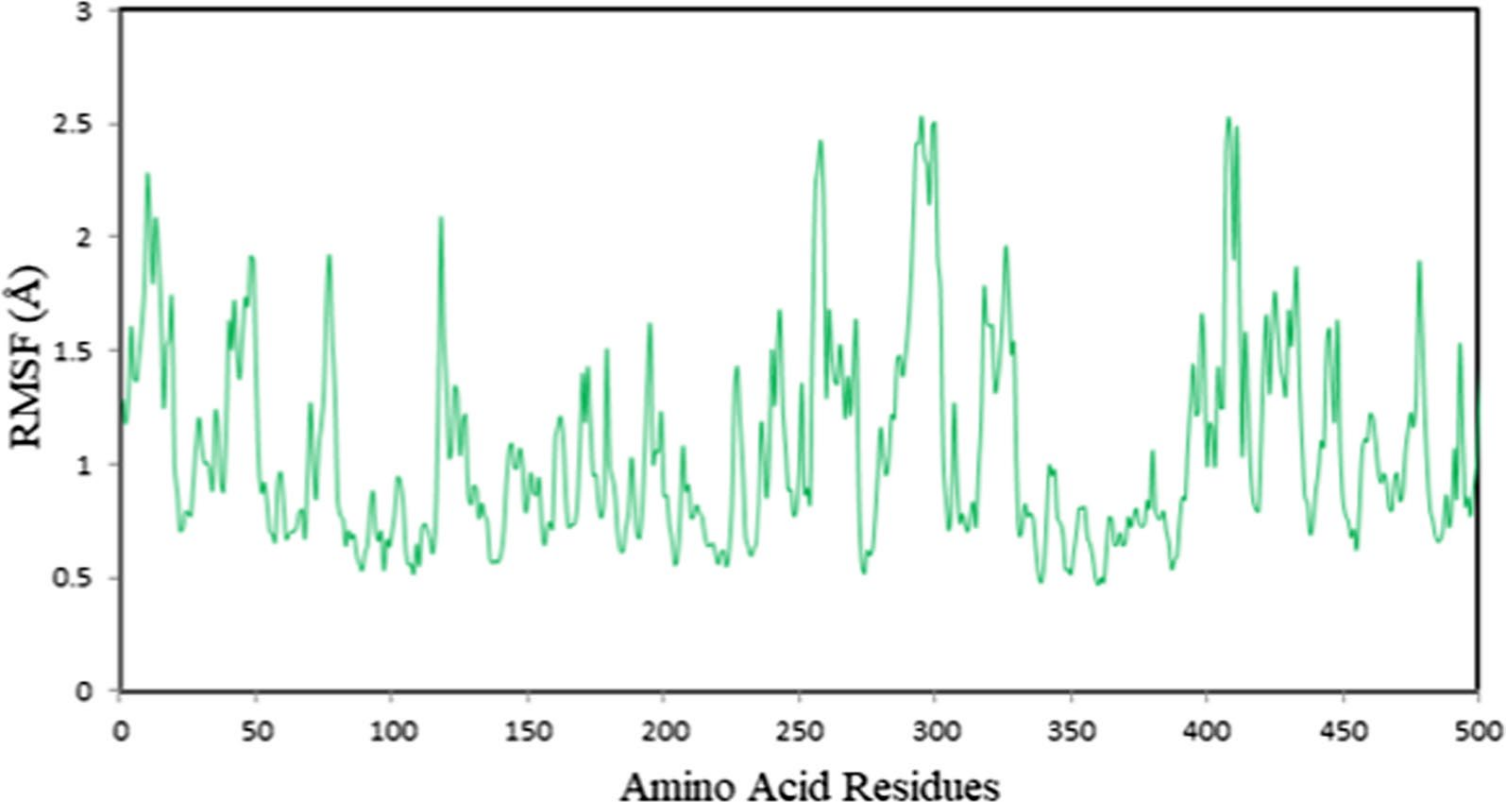
RMSF plot for the protein backbone chain during 100 ns molecular dynamics simulations

The RMSF analysis of the amino acid residues was carried out to gain additional insight regarding protein flexibility. Higher RMSF values imply complex formation instability due to conformational changes, while lower values denote the stability of the amino acid residues. The RMSF plot for the protein residues in Fig. 10 delineated that there were no major fluctuations, and more rigid regions could be detected on the RMSF curve. There were minor fluctuations, as indicated by the RMSF values, which ranged from 0.466 to 2.535 Å. According to this analysis, the protein residues remain rigid and adequate for stable binding.

## Discussion

The use of in silico research has been proven to provide major advantages for drug discovery and design. Drug molecules can be optimized, characterized, and their binding affinities to target proteins improved with the use of well-known in silico computational techniques, including DFT, molecular docking, and dynamics. The DFT technique has provided a chemical and spectroscopic description of the title molecule, which could serve as a database for future studies on related compounds. The studied compound proved superior inhibitory action against the human carbonic anhydrase II enzyme. The compound scored a binding energy in a molecular docking study, which is stronger than other previous finds of comparable compounds against the same enzyme, and also than the standard drugs like Acetazolamide (AZA). The molecular binding modes highlighted the vital role of the sulfonamide group in the interaction with the target protein, promoting more investigations into diarylheterocycle compounds with sulfonamide substitutions rather than sulfonamide-only compounds. ADMET data provides confirmation of a safe profile that advantages the title compound over other approved drugs that have side effects when used to treat carbonic anhydrase. The dynamic interaction with the amino acid residues of the receptor evaluated by molecular dynamics simulation revealed a high stable performance within the protein active site.

## Conclusions

In silico research has proven to be a highly effective tool for drug discovery and development. This study used computer-based analysis to investigate the potential of a new diarylheterocycle compound substituted with sulfonamide as an inhibitor of human carbonic anhydrase (hCA-IIX). Electronic structure and chemical discretion were analyzed using the DFT method with the B3LYP/6-311G (d, p) model. Sulfonamide groups are expected to have the highest reactivity with biological targets since they have the most favored locations for attacks by nucleophiles and electrophiles. Assignments of the IR vibrational bands were determined by comparing the results of the experiment with the calculated data, which indicated sufficient consistency. The UV–vis spectral properties were explored using the TD-DFT in the gas phase to disclose the electronic transition states with the associated absorption wavelength. The stated compound possesses favorable drug-likeness and ADME characteristics. The docking results showed stable bonding interactions via seven strong hydrogen bonds and high binding energy compared to other pre-screened inhibitors. RMSD and RMSF trajectory analysis during a 100 ns molecular dynamics simulation revealed the stable nature of the protein–ligand complex. Therefore, the efficiency of the title compound in inhibiting the hCA-IIX enzyme has been verified by this study, which could be used as a framework for further clinical studies.

## Data Availability

They are available from the corresponding author upon reasonable request.
